# Prenatal vitamin B12 status and cognitive functioning in children at 4 years of age: The ECLIPSES Study

**DOI:** 10.1111/mcn.13580

**Published:** 2023-11-08

**Authors:** Josué Cruz‐Rodríguez, Josefa Canals‐Sans, Carmen Hernández‐Martínez, Núria Voltas‐Moreso, Victoria Arija

**Affiliations:** ^1^ Nutrition and Mental Health Research Group (NUTRISAM) Universitat Rovira i Virgili (URV) Tarragona Spain; ^2^ Institut d'Investigació Sanitària Pere Virgili (IISPV) Tarragona Spain; ^3^ Centre de Recerca en Avaluació i Mesura de la Conducta (CRAMC), Department of Psychology Universitat Rovira i Virgili Tarragona Spain; ^4^ Institut d'Investigació en Atenció Primària IDIAP Jordi Gol Institut Català de la Salut (ICS) Barcelona Spain; ^5^ Collaborative Research Group on Lifestyles, Nutrition and Smoking (CENIT) IDIAP Jordi Gol Reus Spain

**Keywords:** child development, children's health, cognitive functioning, ECLIPSES Study, pregnancy, prenatal nutrition, vitamin B12 levels

## Abstract

Maternal vitamin B12 deficiency has been associated with disturbed cognitive functioning in offspring at different ages during childhood. However, this association has not been explored in pre‐school‐age children. The objective of this study was to examine the association between maternal vitamin B12 levels at the beginning and end of pregnancy and cognitive functioning in their children at 4 years of age. This longitudinal prospective study included a subsample of pregnant women and their children aged 4 years (*n* = 249) who participated in the ECLIPSES Study conducted in the province of Tarragona, Spain, from 2013 to 2017. Maternal vitamin B12 concentrations were determined in the first and third trimesters, and sociodemographic, nutritional and psychological data were collected. The children's cognitive functioning was assessed using the Wechsler Preschool and Primary Scale of Intelligence (WPPSI‐IV) and subtests of the Neuropsychological Assessment of Development (NEPSY‐II). The multivariable models showed a significant relationship between vitamin B12 and the working memory index in the first trimester of the pregnancy but not in the third trimester. Children of mothers in the second vitamin B12 level tertile (314–413 pg/mL) (*β* = 6.468, 95% confidence interval [CI]: = 2.054, 10.882, *p* = 0.004) and third vitamin B12 level tertile (≥414 pg/mL) (*β* = 4.703, 95% CI: = 0.292, 9.114, *p* = 0.037) scored higher in the working memory index of the WPPSI‐IV than the children of mothers with vitamin B12 levels in the first tertile (<314 pg/mL). Maintaining an adequate level of maternal vitamin B12 during early pregnancy contributes to improved performance in childhood working memory at 4 years of age.

## Key messages

- Previous research suggests that maternal vitamin B12 deficiency may negatively affect the neurodevelopment of children at different ages.
- This study analyzes the effect of maternal serum vitamin B12 levels at the beginning and end of pregnancy on cognitive functioning in children at 4 years of age.
- In a population with good socioeconomic conditions and adequate nutritional status, maintaining an adequate level of maternal vitamin B12 during early pregnancy can improve the working memory of their children when they reach 4 years old.
- These findings emphasize the importance of screening for maternal vitamin B12 levels during this critical period of foetal programming to optimize cognitive functioning in offspring. However, further comprehensive and well‐designed long‐term studies are essential to validate and strengthen our results.

## Graphical abstract

Our study found that maintaining an adequate maternal vitamin B12 status during early pregnancy contributes to improved working memory performance at 4 years of age. These findings emphasize the importance of screening for maternal vitamin B12 levels during this critical period to optimize cognitive functioning in offspring.

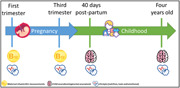

## 1 INTRODUCTION

Maternal nutrition during pregnancy is crucial for the growth and development of the fetus as well as the health and well‐being of the mother. Healthy lifestyle habits and a balanced and adequate diet during this period can help prevent complications such as gestational diabetes, hypertension and premature birth (Cortés‐Albornoz et al., 2021; Mate, 2021). For the fetus, an adequate nutritional status in the mother provides the nutrients needed for the formation of organs and tissues, including the development of the central nervous system, and for the prevention of congenital malformations. Maternal nutritional deficiencies, on the other hand, have been found to increase the risk of altered cognition, as well as other psychological and neuropsychiatric disorders in offspring (Cortés‐Albornoz et al., 2021; Cusick & Georgieff, 2016; England‐Mason & Dewey, 2021).

Neurodevelopment is a complex process that requires a wide range of nutrients for its proper function and development. Some of the most important nutrients for neurodevelopment include iron, fatty acids, zinc, antioxidants, vitamin D and B‐complex vitamins, particularly vitamin B6, vitamin B9 and vitamin B12 (Cortés‐Albornoz et al., 2021; Cusick & Georgieff, 2016; England‐Mason & Dewey, 2021; Iglesias‐Vázquez et al., 2023; Irvine et al., 2023). In particular, vitamin B12 (cobalamin) participates as a cofactor in the one‐carbon metabolism pathway. It is an essential vitamin in the development and maintenance of the central nervous system, as it participates in neurogenesis, neuronal myelination, synaptogenesis and brain growth. Vitamin B12 deficiency during gestation has been associated with impaired neurodevelopment and cognitive functioning (Black, 2008; Molloy et al., 2008; Pavlov et al., 2019).

Vitamin B12 deficiency is a public health problem worldwide, particularly in developing countries and regions where vegetarian diets are prevalent (Green & Miller, 2022; Rashid et al., 2021). In a systematic review on the prevalence of vitamin B12 deficiency conducted by Pawlak et al. (2014), a range of deficiency was identified that reached up to 86.5% in adults and the elderly, 45% in infants, up to 33.3% in children and adolescents, and between 17% and 39% in pregnant women, depending on the trimester of pregnancy. This deficiency can be caused by an inadequate diet or absorption problems and is associated with environmental factors such as smoking, alcohol consumption, lack of physical activity (PA) and low socioeconomic status, which are often linked to limited and less varied diets (John et al., 2023; Shahab‐Ferdows et al., 2015; Sobowale et al., 2022). Additionally, the influence of genetic polymorphisms involved in the metabolism and transport of vitamin B12 and folate can alter vitamin B12 status in mothers (An et al., 2019; Mitchell et al., 2014; Rodríguez‐Carnero et al., 2022).

To the best of our knowledge, eight previous observational studies have assessed the influence of maternal serum vitamin B12 levels on offspring neurodevelopment, and have yielded varying results (Ars et al., 2019; Bhate et al., 2008, 2012; Cruz‐Rodríguez et al., 2023; Keskin et al., 2022; Lai et al., 2019; Veena et al., 2010; Wu et al., 2012). Five of these studies were conducted in Asian countries (Bhate et al., 2008, 2012; Keskin et al., 2022; Lai et al., 2019; Veena et al., 2010), while of the remaining three, two were carried out in Europe (Ars et al., 2019; Cruz‐Rodríguez et al., 2023) and one in Canada (Wu et al., 2012). Among the studies conducted in Asia, Keskin et al. (2022) reported that vitamin B12 deficiency in women during the first trimester of pregnancy is associated with motor, language and social skill problems in 4‐month‐old infants who also had a deficiency of this vitamin (Keskin et al., 2022). The studies conducted by Lai et al. (2019), and Bhate et al. (2012), in 2‐year‐old children found that maternal vitamin B12 deficiency during the third trimester of pregnancy was associated with reduced cognition (Bhate et al., 2012; Lai et al., 2019) and social development (Bhate et al., 2012). Similarly, another study by Bhate et al. (2008), involving 9‐year‐old children, found that children of mothers in the highest vitamin B12 decile during the third trimester of pregnancy performed better in working memory and sustained attention tasks than children of mothers in the lowest vitamin B12 decile. However, Veena et al. (2010) did not find a relationship between maternal vitamin B12 concentrations and cognitive development in children of the same age. The study conducted in Spain reported that the children whose mothers were in the second vitamin B12 tertile during the first trimester of pregnancy had better neonatal performance in motor, language and cognitive skills than the children of mothers in the lowest vitamin B12 tertile (Cruz‐Rodríguez et al., 2023) On the other hand, neither the study conducted in Canada (Wu et al., 2012) with 1.5‐year‐old children nor that conducted in the Netherlands (Ars et al., 2019) with 6–8‐year‐old children found a relationship between maternal levels of vitamin B12 during the second trimester of pregnancy and the cognitive development of the offspring.

Furthermore, the results of four randomized controlled trials (RCTs) conducted in Asian countries (Chandyo et al., 2023; D'souza et al., 2021; Srinivasan et al., 2017; Thomas et al., 2019) were also inconclusive. Two of these trials found that the children of mothers who received vitamin B12 supplements and had high vitamin B12 concentrations in the first trimester (D'souza et al., 2021; Thomas et al., 2019) and third trimesters (Thomas et al., 2019) of pregnancy had higher scores in expressive language at 2 (D'souza et al., 2021) and 2.5 (Thomas et al., 2019) years of age compared with the children of nonsupplemented mothers. However, the studies of Srinivasan et al. (2017), and Chandyo et al. (2023), concluded that vitamin B12 supplementation during pregnancy did not improve the neurodevelopment of the evaluated children at 6 (Chandyo et al., 2023), 9 (Srinivasan et al., 2017) and 12 (Chandyo et al., 2023) months of age.

In summary, the evidence suggests that maternal vitamin B12 deficiency can negatively affect the neurodevelopment of children at different ages (Bhate et al., 2008, 2012; Cruz‐Rodríguez et al., 2023; Keskin et al., 2022; Lai et al., 2019). However, further research is needed to investigate the long‐term effects of maternal vitamin B12 levels on the health and well‐being of their children, as well as the environmental factors that may influence or confound this association (Bhate et al., 2008, 2012; Cruz‐Rodríguez et al., 2023; Keskin et al., 2022; Lai et al., 2019). To date, no information has been published on this association in pre‐school‐age children, a very important stage in the development of higher cognitive functions, and only the study previously published by our research group (Cruz‐Rodríguez et al., 2023) considered a wide range of factors that could influence this association. Based on the above, the present study analyzes the effect of maternal serum vitamin B12 levels at the beginning and end of pregnancy on the cognitive functioning in children at 4 years of age, adjusting for potential associated factors, in healthy pregnant women from the Mediterranean area in Catalonia, Spain.

## 2 MATERIALS AND METHODS

### 2.1 Study design

The ECLIPSES Study (Arija et al., 2014) was an RCT on iron supplementation conducted in the province of Tarragona (Catalonia, Spain) between 2013 and 2017, registered on www.clinicaltrialsregister.eu (number, EUCTR‐2012‐005480‐**) and www.clinicaltrials.gov (number, NCT031968**). A total of 791 participants were recruited during the first prenatal visit from 12 sexual and reproductive health care services (ASSIR) of the Catalan Institute of Health (ICS).

Pregnant women were selected according to the following criteria: healthy women over 18 years of age with less than 12 weeks of gestation and without anaemia (Hb > 110 g/L). Women who had taken iron supplements before the study had a severe (immunosuppression) or chronic disease affecting nutritional status (cancer, diabetes, malabsorption or liver disease), or had a multiple pregnancy were excluded (Arija et al., 2014). They were supplemented with different doses of iron based on their Hb levels at the beginning of the study: those pregnant women with normal‐medium Hb levels (110–130 g/L, Stratum 1) were randomly assigned to receive either 40 or 80 mg/day of iron, while pregnant women normal‐high Hb levels (Hb > 130 g/L, Stratum 2) were randomly assigned to receive either 40 or 20 mg/day of iron (Arija et al., 2014).

In addition to the recruitment visit before Week 12 of gestation, the study included two visits during pregnancy (Weeks 12 and 36), during which vitamin B12 levels in pregnant women were determined. Subsequently, the physical and neurobehavioral development assessment of their children was conducted at 40 days post‐partum and at 4 years of age. The study was designed in accordance with the Helsinki Declarations and was approved by the Ethics Committee of the Institut d'Investigació en Atenció Primaria de Salut (IDIAP) and the Institut d'Investigació Sanitària Pere Virgili (IISPV). Informed consent was obtained from all participating women.

**Figure 1:**
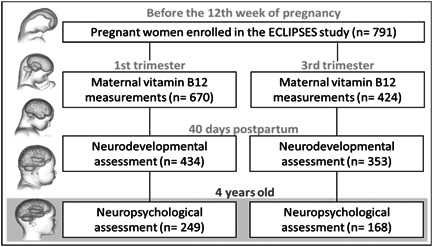
Flowchart of the study.

This analysis is based on a subsample of 249 mothers from the main study whose vitamin B12 levels were recorded in the first and third trimesters of pregnancy and includes an assessment of cognitive functioning in their children at 4 years of age (Figure 1).

### 2.2 Data collection

#### 2.2.1 Maternal

*Obstetrical and sociodemographic data*: Midwives were responsible for collecting the data from the medical records, and the women were visited once in each trimester of pregnancy. A wide range of information was recorded, including maternal age and aspects of medical‐obstetric history such as parity. Maternal weight (in kg to the nearest 0.1 kg) and height (in cm to the nearest 0.1 cm) were measured and the body mass index (BMI) in the first trimester was calculated from these measurements (weight (kg)/height (m)^2^), based on World Health Organization (WHO) criteria (WHO, 2006). Total gestational weight gain (GWG) was the difference between the weights measured during the first‐ and third‐trimester visits.

Information on the education level and occupational status of the participants and their partners was collected using the Catalan Classification of Occupations (CCO‐2011), (Institut d'Estadística de Catalunya–Idescat, 2011), and the family socioeconomic status was calculated as low, medium or high, according to the Hollingshead Index (Hollingshead, 2011).

*Lifestyle habits*: Information collected on maternal lifestyle included dietary habits, PA and smoking. Dietary habits were assessed retrospectively using a semi‐quantitative self‐administered food frequency questionnaire (FFQ) previously validated in the study population (Rodríguez et al., 2008). The FFQ was analyzed by dietitians who calculated daily food intake, energy and nutrient content using the Spanish food‐composition table (Mataix et al., 2009) and the French food‐composition table (Favier et al., 1995). Diet quality was evaluated using a Mediterranean Diet (MedDiet) score (Trichopoulou et al., 2003), based on a scale of ascending values of 0, 1 or 2 points, according to the intake of fruits, vegetables, legumes, cereals, fresh fish, olive oil, meat, dairy products and alcoholic beverages (for alcoholic beverage consumption, nonconsumption scored 2). Participants’ scores ranged from 0 to 18 (higher scores indicate a higher quality diet).

To measure PA, we used the short version of the International Physical Activity Questionnaire (IPAQ‐S), (Craig et al., 2003). The type, frequency and duration of PA performed in a typical week were recorded, and these data were used to obtain metabolic equivalents in minutes per week (MET‐min/week), (Craig et al., 2003). The Fagerström Test (Heatherton et al., 1991) was used to classify the women as smokers or non‐smokers.

*Biochemical data*: Blood samples were collected during Weeks 12 and 36 of gestation. Serum vitamin B12 was determined using the ADVIA Centaur VitB12 immunoassay method. Vitamin B12 levels were classified according to the cutoff point for pregnant women established by the WHO (de Benoist, 2008), with vitamin B12 deficiency as serum levels <200 pg/mL (<150 pmol/mL), marginal vitamin B12 deficiency defined as serum levels 200–300 pg/mL (150–220 pmol/mL), and vitamin B12 sufficiency as serum levels >300 pg/mL (>220 pmol/mL), (de Benoist, 2008). Red blood cell folate (RBC folate) was also determined using the ADVIA immunoassay, and serum ferritin was measured using immunochemiluminescence. We determined the prevalence of RBC folate deficiency (≤340 nmol/L) and depleted ferritin stores (<15 µg/L) using the cutoff points established by the WHO (de Benoist, 2008; World Health Organization‐WHO, 2007).

*Psychological data*: Maternal anxiety status was measured using the State‐Trait Anxiety Inventory (STAI), (Spielberger et al., 1994), which assesses two separate concepts of anxiety: state and trait. Trait evaluates stable and dispositional anxiety, while state assesses situational and transient anxiety. In the present study, only the trait anxiety scores were used.

Parental Intelligence Quotient (IQ) approximation was assessed using the matrix test of the Perceptual Reasoning subscale of the Wechsler Adult Intelligence Scale‐Fourth Edition (WAIS‐IV) administered to both mothers and fathers. The test has a metric scale with a mean of 10 and a standard deviation (SD) of 3 (Wechsler, 2008). When possible, we used the mean from both parents, although when it could only be administered to one of the parents (usually the mother), that score was used.

#### 2.2.2 Infant

*Birth data*: The following data were obtained from hospital obstetric records: sex, gestational age (calculated from the time elapsed since the first day of the last menstrual period), Apgar score and anthropometric measurements at birth (weight, length and head circumference). Mothers were also asked about the feeding method used.

*Psychological data*: The individualized cognitive assessment of the children at 4 years of age was conducted by two trained psychologists using the Spanish version of The Wechsler Preschool and Primary Scale of Intelligence (WPPSI‐IV), (Wechsler, 2014) and the Developmental Neuropsychological Assessment (NEPSY‐II) (Korkman et al., 2007). All children were attending school at the time of the evaluation. The WPPSI‐IV assesses cognitive abilities using 15 subtests, from which five primary indexes, four secondary indexes, and the Full IQ can be obtained. Apart from IQ, the following primary indexes were obtained: Verbal Comprehension Index (VCI), Fluid Reasoning Index (FRI), Working Memory Index (WMI) and Processing Speed Index (PSI). The secondary indexes included were the Vocabulary Acquisition Index (VAI), Nonverbal Index (NVI) and General Ability Index (GAI). All indexes have a mean of 100 and an SD of 15 (Wechsler, 2014).

The NEPSY‐II is a flexible battery of neuropsychological tests designed to assess neurocognitive abilities. In this study, we only used the complementary subtests to the skills assessed with the WPPSI‐IV: verbal fluency (language domain), visuomotor precision (sensorimotor domain) and emotion recognition (social perception domain). The subtests of the NEPSY‐II have a mean of 10 and an SD of 3 (Korkman et al., 2007).

### 2.3 Statistical analyses

Descriptive data were expressed as means or geometric means and standard SD for quantitative variables and as percentages for qualitative variables. The Shapiro–Wilk test was used to test continuous data for normality. After determining the prevalence of vitamin B12 deficiency and marginal vitamin B12 deficiency, the women in the study were divided into three groups (tertiles) categorized as having low, medium or high vitamin B12 levels based on their serum vitamin B12 concentrations. The group with the lowest concentrations (tertile 1) served as the reference category. We used an analysis of variance (ANOVA) test to compare the cognitive development scores of the children across the different maternal vitamin B12 tertiles. Multiple linear regression models were performed using the ENTER method, adjusting for various maternal and child characteristics considered as potential confounding variables due to their demonstrated influence on the cognitive and psychological development of the child (Bhate et al., 2008, 2012; Cruz‐Rodríguez et al., 2023; Keskin et al., 2022; Lai et al., 2019): Maternal age (0: <30 (reference), 1: ≥30); BMI (kg/m2); GWG (kg); education level (0: primary/secondary (reference), 1: university); smoking during pregnancy (0: no (reference), 1: yes); previous parity (0: no (reference), 1: yes); PA during pregnancy (0: low <600 METS‐min/week (reference), 1: moderate/high ≥600 METS‐min/week); diet quality‐MedDiet during pregnancy (score); vitamin B12 intake during pregnancy (µg); folate intake during pregnancy (µg); iron supplementation during pregnancy (40 mg (reference) vs 20 mg and 40 mg (reference) vs. 80 mg); RBC folate levels (nmol/L); serum ferritin (µg/L); mother anxiety during pregnancy (score); parental IQ approximation (score); sex of child (0: male (reference), 1: female); preterm birth (0: no (reference), 1: yes); mode of delivery (0: natural (reference), 1: caesarean); Apgar at 5 min (score); feeding method (0: breastfeeding (reference), 1: formula/mixed); neonatal weight‐height ratio (g/m); and birth head circumference (cm). Given the close physiological connection between vitamin B12, folate and ferritin, we previously analyzed the effect of the interaction of vitamin B12 with these two covariates (RBC folate and ferritin) by including them in the multiple linear regression model. However, both interactions were found to be nonsignificant and did not alter the effect of vitamin B12 on cognitive functioning. Therefore, they were not included in the final model. Additionally, to verify whether average vitamin B12 concentrations have a greater influence on cognitive functioning, a second multiple linear regression model was performed. In this model, the reference category was women with average vitamin B12 levels (314–413 pg/mL, tertile 2) compared with women in the first and third tertiles.

Estimates were presented as a coefficient (β) and 95% confidence intervals (CIs). We assessed multicollinearity by inspecting tolerance values and variance inflation factors (VIF) for this multivariable model. All tolerance values were above 0.4, and all VIF values were below 2.0 (SPSS Statistics, 2021), suggesting no multicollinearity issues, and thus, none of the covariates were eliminated from the models. Statistical significance was set at *p‐*value of <0.05. Statistical analysis was performed using SPSS Statistics software, version 27.0 for Windows (SPSS Inc.).

### 2.4 Ethics statement

The ECLIPSES study was perf ormed in line with the principles of the Declaration of Helsinki and was approved by the Ethical Committee of the Institut d'Investigació en Atenció Primaria de Salut (IDIAP) and the Institut d'Investigació Sanitària Pere Virgili (IISPV) with identification number IISPV 118/2017 (28th September 2017). The ECLIPSES study was registered in the EU Clinical Trial Register, EUCTR‐2012‐005480‐28, and in ClinicalTrials.gov with identification number NCT03196882.

## 3 RESULTS

### 3.1 Characteristics of study participants

A total of 249 mother–infant pairs (52.2% boys, and 47.8% girls) were evaluated. Table 1 shows the sociodemographic, lifestyle and psychological characteristics of the mothers and, WPPSI‐IV and NEPSY‐II scores of the children at 4 years old. The prevalence of marginal vitamin B12 deficiency was 26.1% and deficiency was 2.8% (mean concentration = 375.3 ± 117.6 pg/mL) in the first trimester of pregnancy and 47.6% and 14.9%, respectively (mean concentration = 293.5 ± 108.7 pg/mL), in the third trimester of pregnancy. Only 5.6% of the women presented RBC folate deficiency, and 17.2% had depleted ferritin stores (Table 1).

**Table 1:** Descriptive data of the mother and offspring: sociodemographic, lifestyle, nutrition and psychological (*n* = 249).

*Maternal characteristics*	
Age (years)	31.5 ± 4.6
<30 years, *n* (%)	79 (31.7)
≥30 years, *n* (%)	170 (68.3)
BMI initial (kg/m^2^)	25.0 ± 4.6
Gestational weight gain (kg)	10.3 ± 3.4
Education level, *n* (%)	
Low (primary/secondary)	134 (53.8)
High (university)	115 (46.2)
Smoking during pregnancy, *n* (%)	
No	172 (69.1)
Yes	77 (30.9)
Alcohol consumption during pregnancy, *n* (%)	
No	246 (98.8)
Yes	3 (1.2)
Physical activity during pregnancy (METs/min/week)	3039.5 ± 4600.0
Low (<600 METS/min/week), *n* (%)	66 (26.5)
Moderate/high (≥600 METS/min/week), *n* (%)	183 (73.5)
MedDiet during pregnancy (score)	9.8 ± 2.2
Vitamin B12 intake during pregnancy (µg)	4.3 ± 1.0
Folate intake during pregnancy (µg)	203.3 ± 53.1
Stratum 1 (Hb 110–130 g/L), *n* (%)	
Iron supplementation, 40 mg/day	75 (30.1)
Iron supplementation, 80 mg/day	77 (30.9)
Stratum 2 (Hb > 130 g/L), n (%)	
Iron supplementation, 40 mg/day	47 (18.9)
Iron supplementation, 20 mg/day	50 (20.1)
Previous parity, *n* (%)	
No	112 (45.0)
Yes	137 (55.0)
Parental IQ approximation, (score)	9.2 ± 3.5
Maternal anxiety during pregnancy, (score)	14.4 ± 8.5
Vitamin B12 levels 1st trimester (pg/mL)	375.3 ± 117.6
Vitamin B12 deficiency (<200 pg/mL), *n* (%)	7 (2.8)
Marginal vitamin B12 deficiency (200–300 pg/mL), *n* (%)	65 (26.1)
Tertile 1 (pg/mL)	259.8 ± 38.7
Tertile 2 (pg/mL)	359.3 ± 28.8
Tertile 3 (pg/mL)	506.4 ± 88.7
Vitamin B12 levels 3rd trimester (pg/mL)^a^	293.5 ± 108.7
Vitamin B12 deficiency (<200 pg/mL), *n* (%)^a^	25 (14.9)
Marginal vitamin B12 deficiency (200 to 300 pg/mL), *n* (%)^a^	80 (47.6)
Tertile 1 (pg/mL)	198.5 ± 22.0
Tertile 2 (pg/mL)	269.4 ± 23.3
Tertile 3 (pg/mL)	413.8 ± 101.0
RBC folate levels (nmol/L)	571.1 ± 170.8
RBC folate deficiency (≤340 nmol/L), *n* (%)	14 (5.6)
Serum ferritin levels (µg/L)	42.2 ± 31.9
Depleted ferritin stores (<15 µg/L), *n* (%)	43 (17.2)
*Children characteristics*	
Age (years)	4.0 ± 0.2
Sex, *n* (%)	
Male	130 (52.2)
Female	119 (47.8)
Preterm birth (<37 weeks), *n* (%)	
No	239 (95.9)
Yes	10 (4.1)
Mode of delivery, *n* (%)	
Vaginal delivery	190 (76.3)
Caesarean	59 (23.7)
Feeding method, *n* (%)	
Breastfeeding	190 (76.3)
Mixed feeding/infant formula	60 (24.1)
Birth weight (g)	3294.9 ± 443.1
Birth height (cm)	49.4 ± 2.1
Birth head circumference (cm)	34.6 ± 1.6
Weight‐length ratio neonatal (g/m)	66.5 ± 7.4
Apgar test at 5' (score)	9.9 ± 0.3
WPPSI‐IV scores at 4 years old	
Verbal Comprehension Index, (score)	105.2 ± 12.5
Fluid Reasoning Index, (score)	102.6 ± 13.0
Working Memory Index, (score)	97.7 ± 12.3
Processing Speed Index, (score)	95.9 ± 12.7
Full Intelligence Quotient (IQ), (score)	102.3 ± 11.2
Vocabulary Acquisition Index, (score)	97.5 ± 13.4
Nonverbal Index, (score)	101.0 ± 11.8
General Ability Index, (score)	106.1 ± 11.7
NEPSY‐II scores at 4 years old	
Verbal Fluency, (score)	9.15 ± 2.7
Visual‐Motor Precision, (score)	10.34 ± 3.0
Emotional recognition, (score)	9.20 ± 2.49

*Note*: Values are expressed as a mean ± SD (standard deviation) # or *n* = number (%). Vitamin B12 equivalencies: 200 pg/mL = 150 pmol/mL, 300 pg/mL = 220 pmol/mL. Missing value: MedDiet [*n* = 14(5.6%)]; parental IQ approximation [*n* = 11(4.4%)]; maternal anxiety during pregnancy, [*n* = 21(8.4%)]; weight‐length ratio neonatal, [*n* = 22(8.8%)]; birth head circumference, [*n* = 16(6.4%)]; Apgar test, [*n* = 26(10.4%)].

Abbreviations: BMI, body mass index; Hb, haemoglobin; METs, metabolic equivalent of task; MedDiet, adherence to the Mediterranean diet; NEPPSY‐II, neuropsychological assessment of children; RBC folate, red blood cell folate; WPPSI‐IV, Wechsler Preschool and Primary Scale of Intelligence.

^a^ *n* = 168.

Overall, the mean age of the mothers was 31.5 ± 4.6 years with a mean BMI of 25.0 ± 4.6 kg/m^2^ and a mean GWG of 10.3 ± 3.4 kg. The mean of parental IQ approximation was on par with average (9.2 ± 3.5), 46.2% of the women had a university education, 30.9% were smokers during pregnancy and 26.5% performed low PA during pregnancy. The average MedDiet score observed in our study was 9.8 ± 2.2. Vitamin B12 intake was 4.3 ± 1.0 µg, higher than the recommended intake (2.2 µg), while folate intake was 203.3 ± 53.1 µg, which is below the recommended intake (600 µg) (Table 1). There were no significant differences in most baseline characteristics between the pregnant women who were included in the analysis and those who were not (Supporting Information S1: Table 1).

The anthropometric measurements of the babies at birth were normal (mean weight = 3294.4 ± 443.1 g; mean length = 49.4 ± 2.1; and mean head circumference = 34.6 ± 1.6), the mean Apgar test result at 5 min was 9.9 ± 0.3 points, and 76.3% of the mothers breastfed their infants (Table 1).

No statistically significant differences were found in the WPPSI‐IV and NEPSY‐II scores of the children in relation to maternal vitamin B12 status in the first and third trimesters (Table 2).

**Table 2:** Means of the Wechsler Preschool and Primary Scale of Intelligence (WPPSI‐IV) and Neuropsychological assessment of children (NEPSY‐II) scores at 4 years old according to maternal vitamin B12 concentration tertiles in the first (*n* = 249) and third trimester (*n* = 168) of pregnancy.

Determinants	Vitamin B12 in the first trimester				Vitamin B12 in the third trimester			
	Tertile 1 (<314 pg/mL) *n* = 83	Tertile 2 (314–413 pg/mL) n = 83	Tertile 3 (≥414 pg/mL) *n* = 83	*p* Value	Tertile 1 (<230 pg/mL) *n* = 56	Tertile 2 (230–320 pg/mL) *n* = 56	Tertile 3 (≥321 pg/mL) *n* = 56	*p* Value
	Mean ± SD	Mean ± SD	Mean ± SD		Mean ± SD	Mean ± SD	Mean ± SD	
WPPSI‐IV								
Verbal Comprehension Index	104.20 ± 11.36	106.16 ± 12.47	105.46 ± 13.83	0.599	105.44 ± 11.97	107.95 ± 11.87	106.84 ± 13.92	0.575
Fluid Reasoning Index	103.29 ± 12.80	103.22 ± 11.54	101.45 ± 14.65	0.590	103.35 ± 13.84	101.36 ± 12.60	101.68 ± 13.56	0.698
Working Memory Index	95.85 ± 12.68	98.95 ± 11.87	98.39 ± 12.36	0.225	95.63 ± 11.76	97.36 ± 11.89	99.45 ± 12.46	0.245
Processing Speed Index	97.35 ± 12.19	94.90 ± 13.33	95.50 ± 12.65	0.436	95.89 ± 13.15	96.85 ± 10.37	95.93 ± 13.71	0.900
Full Intelligence Quotient	101.96 ± 10.20	103.10 ± 10.11	101.99 ± 13.18	0.763	102.42 ± 11.73	104.15 ± 10.01	103.39 ± 11.93	0.720
Vocabulary Acquisition Index	97.48 ± 14.37	97.64 ± 11.23	97.56 ± 14.49	0.997	96.30 ± 14.41	98.95 ± 11.23	99.50 ± 14.76	0.410
Nonverbal Index	101.44 ± 10.44	101.19 ± 10.70	100.58 ± 14.20	0.892	100.95 ± 12.39	101.42 ± 12.27	101.20 ± 12.08	0.980
General Ability Index	105.79 ± 10.85	106.99 ± 10.17	105.67 ± 13.94	0.730	106.44 ± 12.36	108.44 ± 10.67	106.43 ± 12.83	0.598
NEPSY‐II								
Verbal Fluency	9.20 ± 2.54	9.14 ± 2.81	9.11 ± 2.76	0.937	8.86 ± 2.54	9.47 ± 2.66	9.64 ± 3.08	0.290
Visual‐Motor Precision	10.65 ± 3.08	9.98 ± 3.02	10.37 ± 3.08	0.363	10.19 ± 3.13	10.40 ± 3.04	11.00 ± 3.28	0.373
Emotional recognition	9.10 ± 2.44	9.46 ± 2.39	9.05 ± 2.62	0.518	9.09 ± 2.70	8.96 ± 2.46	8.98 ± 2.30	0.960

*Note*: Values are expressed in means ± SD (standard deviation). *p*‐values derived from ANOVA in bold type are statistically significant. Vitamin B12 tertiles and equivalencies: 1st trimester (T1 (*n* = 83) < 314 pg/mL (<231.7 pmol/L), T2 (*n* = 83): 314–413 pg/mL (231.7–304.8 pmol/L), and T3 (*n* = 83): ≥414 pg/mL (≥304.8 pmol/L)); 3rd trimester (T1 (*n* = 56) <230 pg/mL (<169.7 pmol/L), T2 (*n* = 56): 230–320 pg/mL (169.7–236.2 pmol/L), and T3 (*n* = 56): ≥321 pg/mL (≥236.2 pmol/L)).

### 3.2 Associations of maternal vitamin B12 levels with cognitive functioning

A first multiple linear regression model, using the first‐trimester vitamin B12 tertiles and adjusted for various environmental factors, showed that the children of mothers in the second tertile (314–413 pg/mL) (*β* = 6.468, 95% CI = 2.054, 10.882, *p* = 0.004) and the third tertile (β = 4.703, 95% CI = 0.292, 9.114, *p* = 0.037) scored higher on the working memory index of the WPPSI‐IV than the children of women with levels of vitamin B12 in the first tertile (reference group). This index also was positively related to the mother's education level (*β* = 7.762, 95% CI = 3.766, 11.758; *p* < 0.001) and negatively related to caesarean birth (*β* = −5.006, 95% CI = −9.487, −0.526, *p* = 0.029) (Table 3). No other significant associations were observed between WPPSI‐IV scores and maternal vitamin B12 levels during the first or third trimester of pregnancy. However, there were associations with other factors such as parental IQ approximation, anxiety, preterm birth and the child's sex (Table 3).

**Table 3:** Multivariate‐adjusted linear regression models of the associations between tertiles of maternal vitamin B12 concentrations in the first (*n* = 184) trimester of pregnancy and the Wechsler Preschool and Primary Scale of Intelligence (WPPSI‐IV) and Neuropsychological assessment of children (NEPSY‐II) scores at 4 years old.

	First trimester		
Determinants	*β*	95% CI	*p* Value
*WPPSI‐IV*			
Verbal Comprehension Index			
Vitamin B12 tertiles (0: T1, 1: T2)	3.092	−1.396, 7.580	0.176
Vitamin B12 tertiles (0: T1, 1: T3)	−0.406	−4.891, 4.079	0.858
Education level (0:primary/secondary, 1:university)	8.310	4.246, 12.373	<0.001
Sex of child (0:male, 1:female)	3.780	0.073, 7.487	0.046
	*R* ^2^ = 0.183, *F* = 25,158 = 2.63, *p* = <0.001		
Fluid Reasoning Index			
Vitamin B12 tertiles (0: T1, 1: T2)	−1.037	−5.699, 3.626	0.661
Vitamin B12 tertiles (0: T1, 1: T3)	−5.367	−10.027, −0.708	0.024
	*R* ^2^ = 0.050, *F* = 25,158 = 1.38, *p* = 0.118		
Working Memory Index			
Vitamin B12 tertiles (0: T1, 1: T2)	6.468	2.054, 10.882	**0.004**
Vitamin B12 tertiles (0: T1, 1: T3)	4.703	0.292, 9.114	**0.037**
Education level (0:primary/secondary, 1:university)	7.762	3.766, 11.758	**<0.001**
Mode of delivery (0:normal, 1:caesarean)	−5.006	−9.487, −0.526	**0.029**
	*R* ^2^ = 0.114, *F* = 25,158 = 1.93, ***p*** = **0.008**		
Processing Speed Index			
Vitamin B12 tertiles (0: T1, 1: T2)	−2.683	−7.265, 1.900	0.249
Vitamin B12 tertiles (0: T1, 1: T3)	−1.301	−5.880, 3.278	0.576
Education level (0:primary/secondary, 1:university)	4.640	0.491, 8.789	0.029
Maternal anxiety during pregnancy (score)	−0.266	−0.503, −0.030	0.028
Sex of child (0:male, 1:female)	7.207	3.422, 10.992	<0.001
	*R* ^2^ = 0.123, *F* = 25,158 = 2.02, *p* = 0.005		
Full Intelligence Quotient (IQ)			
Vitamin B12 tertiles (0: T1, 1: T2)	1.425	−2.622, 5.472	0.488
Vitamin B12 tertiles (0: T1, 1: T3)	−1.879	−5.923, 2.166	0.360
Education level (0:primary/secondary, 1:university)	6.598	2.934, 10.263	<0.001
	*R* ^2^ = 0.130, *F* = 25,158 = 2.09, *p* = 0.003		
Vocabulary Acquisition Index			
Vitamin B12 tertiles (0: T1, 1: T2)	1.548	−3.154, 6.249	0.516
Vitamin B12 tertiles (0: T1, 1: T3)	−0.856	−5.554, 3.842	0.719
Education level (0:primary/secondary, 1:university)	5.423	1.166, 9.680	0.013
Parental IQ approximation (score)	1.244	0.622, 1.865	<0.001
Sex of child (0:male, 1:female)	6.051	2.168, 9.935	0.002
	*R* ^2^ = 0.197, *F* = 25,158 = 2.79, *p* = <0.001		
Nonverbal Index			
Vitamin B12 tertiles (0: T1, 1: T2)	0.174	−4.154, 4.502	0.937
Vitamin B12 tertiles (0: T1, 1: T3)	−2.672	−6.996, 1.653	0.224
	*R* ^2^ = 0.033, *F* = 25,158 = 1.25, *p* = 0.203		
General Ability Index			
Vitamin B12 tertiles (0: T1, 1: T2)	1.200	−2.932, 5.331	0.567
Vitamin B12 tertiles (0: T1, 1: T3)	−2.960	−7.089, 1.168	0.159
Education level (0:primary/secondary, 1:university)	5.692	1.952, 9.433	0.003
Parental IQ approximation (score)	0.759	0.213, 1.305	0.007
Preterm birth (0:no, 1:yes)	−13.200	−23.893, −2.508	0.016
	*R* ^2^ = 0.165, *F* = 25,158 = 2.44, *p* = <0.001		
*NEPSY‐II*			
Verbal Fluency			
Vitamin B12 tertiles (0: T1, 1: T2)	0.181	−0.843, 1.206	0.727
Vitamin B12 tertiles (0: T1, 1: T3)	−0.617	−1.640, 0.407	0.236
Birth head circumference (cm)	−0.386	−0.673, −0.098	0.009
	*R* ^2^ = 0.087, *F* = 25,158 = 1.69, *p* = 0.028		
Visual‐Motor Precision			
Vitamin B12 tertiles (0: T1, 1: T2)	−0.324	−1.531, 0.883	0.596
Vitamin B12 tertiles (0: T1, 1: T3)	−0.026	−1.233, 1.180	0.966
	*R* ^2^ = −0.002, *F* = 25,158 = 0.98, *p* = 0.492		
Emotional recognition			
Vitamin B12 tertiles (0: T1, 1: T2)	0.782	−0.102, 1.666	0.083
Vitamin B12 tertiles (0: T1, 1: T3)	−0.036	−0.919, 0.848	0.936
WAIS, mother IQ (score)	0.159	0.042, 0.276	0.008
Preterm birth (0:no, 1:yes)	−2.571	−4.859, −0.283	0.028
Mode of delivery (0:normal, 1:caesarean)	1.066	0.168, 1.963	0.020
Sex of child (0:male, 1:female)	1.499	0.769, 2.229	<0.001
	*R* ^2^ = 0.125, *F* = 25,158 = 2.04, *p* = 0.004		

*Note*: Bold value indicate statistical significance at *p* < 0.05.

Abbreviations: BMI, body mass index; MedDiet, Mediterranean diet; RBC folate, red blood cell folate.

Models were performed adjusting for the following variables: vitamin B12 tertiles at 1st trimester (T1 (*n* = 83), reference: <314 pg/mL (<231.7 pmol/L), T2 (*n* = 83): 314–413 pg/mL (231.7–304.8 pmol/L), and T3 (*n* = 83): ≥414 pg/mL (≥304.8 pmol/L)); maternal age (0: <30, 1: ≥30), BMI (kg/m^2^); gestational weight gain (kg); education level (0:primary/secondary, 1:university); smoking during pregnancy (0:no, 1:yes); previous parity (0:no, 1:yes); physical activity during pregnancy (0:low <600 METS‐min/week, 1:moderate/high ≥600 METS‐min/week); diet quality‐MedDiet during pregnancy(score); vitamin B12 intake during pregnancy (µg); folate intake during pregnancy (µg); iron supplementation during pregnancy (40 mg (reference) vs. 20 mg and 40 mg (reference) vs. 80 mg); RBC folate levels (nmol/L); serum ferritin (µg/L); maternal anxiety during pregnancy (score); parental IQ approximation (score); sex of child (0:male, 1:female); preterm birth (0:no, 1:yes); mode of delivery (0:normal, 1:caesarean); Apgar at 5 min (score); feeding method (0:breastfeeding, 1:formula/mixed); neonatal weight‐height ratio (g/m); birth head circumference (cm). The model was obtained using the ENTER method. Values are expressed as a coefficient beta (β) and confidence interval (95% CI).

The results were consistent with those of a second multiple linear regression model in which women in the second tertile of serum vitamin B12 concentrations were used as a reference. The analysis showed that children of mothers in the first (*β* = −6.468, 95% CI = −10.882, −2.054; *p* = 0.004) scored lower on the working memory index than children of mothers in the second tertile and that the children of mothers in the third tertile do not achieve better scores compared with mothers in the second tertile (*β* = −1.765, 95% CI = −6.286, 2.756; *p* = 0.442), (model: R^2^ = 0.114, *F* = 25,158 = 1.93, *p* = 0.008).

We did not find any significant associations between the neurocognitive skills evaluated by means of the NEPSY‐II and maternal serum vitamin B12 levels in any of the trimesters evaluated. We also conducted another multiple linear regression analysis with the continuous vitamin B12 values using the same adjustment variables. However, no effects were observed on any of the neurodevelopmental index (Supporting Information S1: Table 2). However, there were significant associations between neurocognitive skills and other factors, such as the parental IQ approximation, premature birth, delivery mode, head circumference at birth and the sex of the child (Table 3).

## 4 DISCUSSION

To our knowledge, this is the first study that has examined the association between maternal vitamin B12 levels during pregnancy and cognitive abilities at 4 years of age. Our study, conducted on a sample of mother–child pairs from the Mediterranean region of Spain, found that, although children's mean WPPSI‐IV scores were within the normal range, a sufficient level of maternal serum vitamin B12 during the first trimester of pregnancy was an independent predictor of cognitive performance, specifically working memory at 4 years of age, after controlling for a number of potential confounders.

When examining the relationship between maternal vitamin B12 levels and offspring cognitive functioning, we considered both continuous values and tertiles of maternal vitamin B12. However, we only found significant effects in analyzes using tertiles of maternal vitamin B12 levels. In this analysis by tertiles, the cut‐off point of our first tertile (<314 pg/mL) was very close to the normality threshold defined by the WHO (>300 pg/mL) (de Benoist, 2008), which able to differentiate two ranges of normality: medium (second tertile) and medium‐high (third tertile). This differentiation interested us since in a previous study by our research group we observed that there was a different level of protective effect on the neurodevelopment of the child at 40 days of age between these two categories (Cruz‐Rodríguez et al., 2023).

Our results indicate, that maternal vitamin B12 levels in the second and third tertiles have a positive effect on working memory compared with levels maternal vitamin B12 in the first tertile [314–413 pg/mL, tertile 2 (*β* = 6.468) and ≥414 pg/mL, tertile 3 (*β* = 4.703)], with a greater effect observed in children of mothers in the second tertile; this suggests that the effect of maternal vitamin B12 on child working memory does not appear to follow a linear relationship. To our knowledge, a physiological mechanism has not yet been identified to explain why higher levels of vitamin B12 within normal ranges do not appear to have a superior cognitive impact compared with intermediate levels. It is plausible that there is a threshold effect in which medium‐high levels of vitamin B12 exceed the necessary requirements for optimal development of the baby's nervous system. Once this optimal level is exceeded, additional levels of vitamin B12 are likely to have no additional impact on the baby's neurodevelopment. Furthermore, even in situations where the mother has very high levels of vitamin B12, the fetus could actively regulate its absorption or utilization of vitamin B12 to keep it within an optimal range for its development, since it only accumulates between 0.1 and 0.2 µg/day (Allen, 2002). However, to gain a more complete understanding of this relationship and how vitamin B12 levels affect cognitive function, more research is needed to identify thresholds of benefit.

Working memory is governed by the dorsolateral prefrontal cortex and is a crucial component of executive functioning. It establishes a fundamental link between perception, attention, memory and action. This brain system temporarily stores multiple memories and allows for the manipulation of information required for complex cognitive tasks. In the WPPSI‐IV, the WMI is made up of two visual processing tests (recognition and localization), which require the activation of other neuroanatomical structures such as the occipital lobe (Bree & Beljan, 2016; Ebert et al., 2023). Working memory has also been found to play an important role in child development and is related to academic performance, reading comprehension, mathematical skills and language development (Cortés Pascual et al., 2019; Quílez‐Robres et al., 2021). In addition, it has been associated with neurodevelopmental disorders such as attention deficit hyperactivity disorder (ADHD), (Irwin et al., 2021) and schizophrenia (Zhou et al., 2022).

The available evidence on the effect of maternal levels of vitamin B12 on the neurodevelopment of offspring suggests that maternal deficiency of this vitamin negatively affects the neurodevelopment of children at different ages (Bhate et al., 2008, 2012; Cruz‐Rodríguez et al., 2023; D'souza et al., 2021; Keskin et al., 2022; Lai et al., 2019; Thomas et al., 2019). Two RCTs of maternal vitamin B12 supplementation, conducted in India, reported that children born to mothers who received supplements and had higher levels of this vitamin during the first and third trimesters of pregnancy showed better cognitive development at 2 years of age compared with children of mothers who did not receive supplements (D'souza et al., 2021; Thomas et al., 2019). For their part, observational studies have reported found that Indian children (9 years of age) of mothers with low serum vitamin B12 levels during the third trimester of pregnancy had lower performance in sustained attention (evaluated with the Colour Trails Test) and working memory (evaluated with the Digit Span Backward Task) compared with children of mothers with high serum vitamin B12 levels. In contrast, no effects on neuropsychological functioning were found in another study that also evaluated the third trimester of pregnancy in the Indian population (Veena et al., 2010) or in one that assessed the second trimester in the Dutch population (Ars et al., 2019). In this regard, we detected an effect on working memory during the first trimester of pregnancy, but not during the third trimester, even though the vitamin B12 levels were lower in this period. Also, previous studies on maternal vitamin B12 status during pregnancy have reported that deficiency during the first trimester of pregnancy may negatively impact children's neurodevelopment at 1 month (Cruz‐Rodríguez et al., 2023) and 4 months of age (Keskin et al., 2022). This association between child working memory and maternal vitamin B12 status in the first trimester is supported by the crucial role of vitamin B12 in early processes of central nervous system development, such as neurogenesis, which primarily occurs at the beginning of pregnancy (Black, 2008; Molloy et al., 2008). Therefore, alterations in neurological development are more closely related to the period when the fetus needs more vitamin B12 and is most vulnerable to deficiency (Cruz‐Rodríguez et al., 2023). Hence, it is possible that previous studies that found that maternal vitamin B12 deficiency during the third trimester impacted neurodevelopment (Bhate et al., 2008; Bhate et al., 2012; Lai et al., 2019) would have yielded the same results for first‐trimester deficiency had they evaluated it.

In line with other studies, we found that several other factors influence neuropsychological development, such as maternal education level (Joseph et al., 2018; Stiver et al., 2015) and parental IQ (Lean et al., 2018; Ronfani et al., 2015), type of delivery (Keag et al., 2018; Takács et al., 2021), head circumference (Bhate et al., 2008; Koshy et al., 2021; Kirkegaard et al., 2020) and child sex (Saylik et al., 2018; Voyer et al., 2021). Maternal educational attainment is a significant predictor of neurodevelopment in children (Joseph et al., 2018; Stiver et al., 2015). This association can be attributed not only to genetic factors influencing cognitive abilities, such as IQ, but also to greater cognitive stimulation of the children and the socioeconomic advantages associated with both higher maternal education and higher family socioeconomic status, including improved living conditions and greater access to essential resources such as healthcare and nutritious food (Stiver et al., 2015). Specifically, Rosen et al. (2020) found a relationship between executive function performance in children of 5–6 years and early cognitive stimulation mediated by socioeconomic status. In addition, in accordance with our data, other authors have observed that higher maternal IQ is associated with better performance on intelligence tests, cognitive abilities and academic achievement in children IQ (Lean et al., 2018; Ronfani et al., 2015).

The effect of delivery mode on neurodevelopment has been the focus of several studies. Some studies suggest that caesarean section delivery may adversely impact the child's neurodevelopment compared with vaginal delivery due to increased venous pressure, modifications of the hypothalamic‐pituitary‐adrenal axis, and altered colonization of the intestinal microbiota (Keag et al., 2018; Takács et al., 2021). For example, alterations in memory have been observed (Sznajder et al., 2022) as well as disorders in verbal and non‐verbal development and general intelligence (González‐Valenzuela et al., 2019).

Head circumference is a robust indicator of childhood neurodevelopment and provides a dynamic view of overall brain growth and internal structures (Bhate et al., 2008; Koshy et al., 2021; Kirkegaard et al., 2020). Several studies have demonstrated a relationship between head size, measured by head circumference, and aspects such as verbal cognition, cognitive performance and IQ (Koshy et al., 2021; Kirkegaard et al., 2020). The sex of the child also seems to affect neurodevelopment. Several studies have concluded that emotional and cognitive functions are indeed sensitive to sex differences (Saylik et al., 2018; Voyer et al., 2021). The use of cerebral hemispheres plays an important role in this aspect: Girls tend to use both hemispheres more frequently during the early years of life, which gives them a broader scope of memory and a greater ability to multitask, while boys tend to focus on single tasks and activities that primarily involve one cerebral hemisphere (Saylik et al., 2018; Turano et al., 2019; Voyer et al., 2021).

This study has various strengths based on several aspects. First, the original design of the study was a triple‐blind, population‐based RCT (Arija et al., 2014), providing a high level of scientific rigour and variable control. Second, a wide range of data was collected from the mothers, including sociodemographic, clinical, emotional and lifestyle information. This included a comprehensive and detailed assessment of children's neuropsychological functioning using internationally validated tests adapted to the Spanish population, ensuring the reliability of the results. Furthermore, this is the first study to evaluate the effect of maternal vitamin B12 levels at two different stages of pregnancy on the neurological development of the offspring at 4 years of age, while also considering a wide range of confounding factors.

However, some limitations should also be mentioned. For instance, there was a significant loss of sample at 4 years compared with the evaluation in the first trimester of pregnancy, which may have affected the statistical accuracy. Nevertheless, selection bias was considered by comparing the characteristics of included and nonincluded participants in this analysis. Another limitation was the lack of additional biomarkers for vitamin B12. Since serum vitamin B12 determination reflects late‐stage deficiency, the inclusion of other biomarkers such as methylmalonic acid, holotranscobalamin or homocysteine, which are early indicators of deficiency, would have enriched the assessment and provided a more accurate perspective on the participants’ vitamin B12 levels (Al‐Musharaf, et al., 2020; Obeid et al., 2019). Serum vitamin B12 levels were also not measured in the pre‐school‐aged children at the time of the psychological evaluation.

## 5 CONCLUSIONS

Our study found that maintaining an adequate maternal vitamin B12 status during early pregnancy contributes to improved working memory performance at 4 years of age. These findings emphasize the importance of screening for maternal vitamin B12 levels during this critical period of foetal programming to optimize cognitive functioning in offspring. However, to validate and strengthen our results, further comprehensive and well‐designed long‐term studies are essential.

## AUTHOR CONTRIBUTIONS

Victoria Arija designed the research. Victoria Arija and Josefa Canals‐Sans conducted the research. Carmen Hernández‐Martínez and Núria Voltas‐Moreso conducted the psychological assessments. Josué Cruz‐Rodríguez and Josefa Canals‐Sans analyzed the data and wrote the article. All authors revised the manuscript for important intellectual content and read and approved the final manuscript. Victoria Arija is the guarantor of this work and, as such, had full access to all the data in the study and takes responsibility for the integrity of the data and the accuracy of the data analysis.

## CONFLICT OF INTEREST STATEMENT

The authors declare no conflict of interest.

## Supporting information

Supporting information.

## Data Availability

Correspondence and requests for materials should be addressed to VA.
