# Supplementary material for: Prenatal vitamin B12 status and cognitive functioning in children at 4 years of age: The ECLIPSES Study
Source: Matern Child Nutr. 2023 Nov 8;20(1):e13580. doi: 10.1111/mcn.13580 (PMC10750008; doi:10.1111/mcn.13580)
Supplement: Supplementary file 1 — Supporting information. [file MCN-20-e13580-s001.docx]

**Prenatal vitamin B12 status and** **cognitive functioning in children at four years of age: The ECLIPSES Study**

**Supplementary Table 1** Maternal characteristics of participants included and not included in the present analysis.

| **Maternal characteristics** | Included  (n=249) | Not included  (n=542) | ***p*-Value** |
| --- | --- | --- | --- |
| Age (years) | 31.5 ± 4.6 | 29.7 ± 5.2 | <0.001 |
| < 30 years, n (%) | 79 (31.7) | 249 (45.9) | <0.001 |
| ≥ 30 years, n (%) | 170 (68.3) | 293 (54.1) |  |
| BMI initial (kg/m^2^) | 25.0 ± 4.6 | 25.0 ± 4.4 | 0.955 |
| Gestational weight gain (kg) | 10.3 ± 3.4 | 10.3 ± 3.8 | 0.850 |
| Education level, n (%) |  |  |  |
| Low (primary/secondary) | 134 (53.8) | 430 (79.3) | <0.001 |
| High (university) | 115 (46.2) | 112 (20.7) |  |
| Smoking during pregnancy, n (%) |  |  |  |
| No | 172 (69.1) | 361 (66.6) | 0.514 |
| Yes | 77 (30.9) | 181 (33.4) |  |
| Alcohol consumption during pregnancy, n (%) |  |  |  |
| No | 246 (98.8) | 539 (99.4) | 0.386 |
| Yes | 3 (1.2) | 3 (0.6) |  |
| Physical activity during pregnancy (METs/min/week) | 3039.5 ± 4600.0 | 3347.9 ± 4657.2 | 0.386 |
| MedDiet during pregnancy (score) | 9.8 ± 2.2 | 9.6 ± 2.1 | 0.236 |
| Vitamin B12 intake during pregnancy (µg) | 4.3 ± 1.0 | 4.4 ± 1.2 | 0.157 |
| Folate intake during pregnancy (µg) | 203.3 ± 53.1 | 201.4 ± 63.9 | 0.704 |
| Previous parity, n (%) |  |  |  |
| No | 112 (45.0) | 203 (37.5) | 0.051 |
| Yes | 137 (55.0) | 338 (62.5) |  |
| Maternal anxiety during pregnancy, (score) | 14.4 ± 8.5 | 12.8 ± 8.1 | 0.017 |
| Vitamin B12 levels 1^st^ trimester (pg/mL) | 375.3 ± 117.6 | 361.5 ± 117.4 | 0.140 |
| Marginal vitamin B12 deficiency (200 to 300 pg/mL), n (%) | 104 (41.8) | 172 (37.6) | 0.752 |
| Vitamin B12 deficiency (< 200 pg/mL), n (%) | 29 (11.6) | 67 (14.6) |  |
| Vitamin B12 levels 3^rd^ trimester (pg/mL) | 293.5 ± 108.7 | 291.3 ± 116.8 | 0.850 |
| Marginal vitamin B12 deficiency (200 to 300 pg/mL), n (%) | 80 (47.6) | 110 (43.5) | 0.221 |
| Vitamin B12 deficiency (< 200 pg/mL), n (%) | 25 (14.9) | 54 (21.3) |  |
| RBC folate levels (nmol/L) | 571.1 ± 170.8 | 533.0 ± 207.1 | 0.012 |
| Serum ferritin levels (µg/L) | 42.2 ± 31.9 | 41.6 ± 29.0 | 0.774 |

Values are expressed as a mean ± SD (standard deviation) # or n=number (%). Abbreviations: BMI, body mass index; METs, metabolic equivalent of task; MedDiet, adherence to the Mediterranean diet; RBC folate, red blood cell folate. Vitamin B12 equivalencies: 200 pg/mL = 150 pmol/mL, 300 pg/mL = 220 pmol/mL. Missing value: MedDiet [n = 61(7.7%)]; vitamin B12 intake during pregnancy [n = 61(7.7%)]; folate intake during pregnancy [n = 61(7.7%)]; maternal anxiety during pregnancy, [n = 89(11.25%)]; vitamin B12 levels 1st^t^ trimester [n = 121(15.2%)]; vitamin B12 levels 3rd trimester [n = 370(46.7%)]; RBC folate levels, [n = 229(28.9%)].

**Suplementary Table 2** Multivariate-adjusted linear regression models of the associations between levels of maternal vitamin B12 concentrations in the first (n=184) trimester of pregnancy and the Wechsler Preschool and Primary Scale of Intelligence (WPPSI-IV) and Neuropsychological assessment of children (NEPSY-II) scores at 4 years old.

|  | **First trimester** | | |
| --- | --- | --- | --- |
| **Determinants** | **β** | **95% CI** | ***p*-Value** |
| **WPPSI-IV** |  |  |  |
| **Verbal Comprehension Index** |  |  |  |
| Vitamin B12 (pg/dL) | -0.004 | -0.020, 0.012 | 0.657 |
| Education level (0:primary/secondary, 1:university) | 8.297 | -4.199, 12.396 | <0.001 |
|  | R2= 0.283, F=24,159=2.61, p=<0.001 | | |
| **Fluid Reasoning Index** |  |  |  |
| Vitamin B12 (pg/dL) | -0.017 | -0.033, 0.000 | 0.058 |
|  | R^2^= 0.171, F=24,159=1.36, p=0.131 | | |
| **Working Memory Index** |  |  |  |
| Vitamin B12 (pg/dL) | 0.010 | -0.006, 0.026 | 0.226 |
| Education level (0:primary/secondary, 1:university) | 7.597 | 3.504, 11.690 | < 0.001 |
|  | R^2^= 0.050, F=24,159=1.63, p=0.039 | | |
| **Processing Speed Index** |  |  |  |
| Vitamin B12 (pg/dL) | -0.004 | -0.020, 0.012 | 0.616 |
| Education level (0:primary/secondary, 1:university) | 4.749 | 0.583, 8.914 | 0.026 |
| Maternal anxiety during pregnancy (score) | -0.265 | -0.501, -0.028 | 0.028 |
| Sex of child (0:male, 1:female) | 7.310 | 3.504, 11.115 | < 0.001 |
|  | R^2^= 0.237, F=24,159=2.06, p=0.005 | | |
| **Full Intelligence Quotient (IQ)** |  |  |  |
| Vitamin B12 (pg/dL) | -0.007 | -0.021, 0.008 | 0.356 |
| Education level (0:primary/secondary, 1:university) | 6.630 | 2.944, 10.316 | <0.001 |
|  | R^2^= 0.242, F=24,159=2.11, p=0.003 | | |
| **Vocabulary Acquisition Index** |  |  |  |
| Vitamin B12 (pg/dL) | -0.008 | -0.025, 0.008 | 0.314 |
| Education level (0:primary/secondary, 1:university) | 5.558 | 1.299, 9.818 | 0.011 |
| Parental IQ approximation (score) | 1.217 | 0.606, 1.828 | <0.001 |
| Sex of child (0:male, 1:female) | 5.638 | 1.747, 9.529 | 0.005 |
|  | R^2^= 0.307, F=24,159=2.93, p=<0.001 | | |
| **Nonverbal Index** |  |  |  |
| Vitamin B12 (pg/dL) | -0.007 | -0.022, 0.008 | 0.359 |
|  | R^2^= 0.159, F=24,159=1.25, p=0.205 | | |
| **General Ability Index** |  |  |  |
| Vitamin B12 (pg/dL) | -0.011 | -0.26, 0.004 | 0.143 |
| Education level (0:primary/secondary, 1:university) | 5.780 | 2.015, 9.544 | 0.003 |
| Parental IQ approximation (score) | 0.693 | 0.152, 1.233 | 0.012 |
| Preterm birth (0:no, 1:yes) | -14.836 | -25.361, -4.312 | 0.006 |
|  | R^2^= 0.271, F=24,159=2.46, p=<0.001 | | |
| **NEPSY-II** |  |  |  |
| **Verbal Fluency** |  |  |  |
| Vitamin B12 (pg/dL) | -0.002 | -0.005, 0.002 | 0.347 |
| Birth head circumference (cm) | -0.372 | -0.659, -0.085 | 0.011 |
|  | R^2^= 0.204, F=24,159=1.69, p=0.030 | | |
| **Visual-Motor Precision** |  |  |  |
| Vitamin B12 (pg/dL) | 0.002 | -0.002, 0.006 | 0.329 |
|  | R^2^= -0.138, F=24,159=1.06, p=0.395 | | |
| **Emotional recognition** |  |  |  |
| Vitamin B12 (pg/dL) | -0.001 | -0.004, 0.003 | 0.690 |
| WAIS, mother IQ (score) | 0.145 | 0.028, 0.261 | 0.015 |
| Preterm birth (0:no, 1:yes) | -2.847 | -5.114, -0.580 | 0.014 |
| Mode of delivery (0:normal, 1:caesarean) | 1.179 | 0.280, 2.078 | 0.010 |
| Sex of child (0:male, 1:female) | 1.427 | 0.686, 2.168 | < 0.001 |
|  | R^2^= 0.225, F=24,159=1.92, p=0.009 | | |

Models were performed adjusting for the following variables: vitamin B12 levels; maternal age (0: <30, 1: ≥30), BMI (kg/m^2^); gestational weight gain (kg); education level (0:primary/secondary, 1:university); smoking during pregnancy (0:no, 1:yes); previous parity (0:no, 1:yes); physical activity during pregnancy (0:low <600 METS-min/week, 1:moderate/high ≥ 600 METS-min/week); diet quality-MedDiet during pregnancy(score); vitamin B12 intake during pregnancy (µg); folate intake during pregnancy (µg); iron supplementation during pregnancy (40 mg (reference) vs 20 mg and 40 mg (reference) vs 80 mg); RBC folate levels (nmol/L); serum ferritin (µg/L); maternal anxiety during pregnancy (score); parental IQ approximation (score); sex of child (0:male, 1:female); preterm birth (0:no, 1:yes); mode of delivery (0:normal, 1:caesarean); Apgar at 5 minutes (score); feeding method (0:breastfeeding, 1:formula/mixed); neonatal weight-height ratio (g/m); birth head circumference (cm). The model was obtained using the ENTER method. Values are expressed as a coefficient beta (β) and confidence interval (95% CI). Statistical significance (*p*-value <0.05). Abbreviations: BMI, body mass index; MedDiet, Mediterranean diet; RBC folate, red blood cell folate.
